# The web of silence: a qualitative case study of early intervention and support for healthcare workers with mental ill-health

**DOI:** 10.1186/1471-2458-14-138

**Published:** 2014-02-08

**Authors:** Sandra E Moll

**Affiliations:** 1 School of Rehabilitation Science, McMaster University, Institute for Applied Health Science, 4th Floor, 1400 Main St. W., Hamilton ON L8S 1C7, Canada

**Keywords:** Healthcare workers, Workplace mental health, Stigma, Organizational research, Early intervention, Mental illness, Qualitative methods

## Abstract

**Background:**

There is a high rate of stress and mental illness among healthcare workers, yet many continue to work despite symptoms that affect their performance. Workers with mental health issues are typically ostracized and do not get the support that they need. If issues are not addressed, however, they could become worse and compromise the health and safety, not only of the worker, but his/her colleagues and patients. Early identification and support can improve work outcomes and facilitate recovery, but more information is needed about how to facilitate this process in the context of healthcare work. The purpose of this study was to explore the key individual and organizational forces that shape early intervention and support for healthcare workers who are struggling with mental health issues, and to identify barriers and opportunities for change.

**Methods:**

A qualitative, case study in a large, urban healthcare organization was conducted in order to explore the perceptions and experiences of employees across the organization. In-depth interviews were conducted with eight healthcare workers who had experienced mental health issues at work as well as eight workplace stakeholders who interacted with workers who were struggling (managers, coworkers, union leaders). An online survey was completed by an additional 67 employees. Analysis of the interviews and surveys was guided by a process of interpretive description to identify key barriers to early intervention and support.

**Results:**

There were many reports of silence and inaction in response to employee mental health issues. Uncertainty in identifying mental health problems, stigma regarding mental ill health, a discourse of professional competence, social tensions, workload pressures, confidentiality expectations and lack of timely access to mental health supports were key forces in preventing employees from getting the help that they needed. Although there were a few exceptions, the overall study findings point to many barriers to supporting employees with mental health issues.

**Conclusions:**

In order to address the complex knowledge, attitudinal, interpersonal and organizational barriers to action, a multi-layered knowledge translation strategy is needed, that considers not only mental health literacy and anti-stigma interventions, but addresses the unique context of the work environment that can act as a barrier to change.

## Background

Healthcare workers in many industrialized countries report high levels of workplace stress and are at a higher risk of mental health problems than other occupational groups [1-3]. Healthcare is characterized by emotionally demanding work in an environment that is often pressured by fiscal restraint leading to high workloads, insufficient staffing, and increased social tensions [4]. One of the consequences of workplace stress is absenteeism; healthcare workers are more likely to miss work due to illness or disability and tend to be absent for significantly more days than workers in other sectors [5]. Another consequence is presenteeism; a high proportion of healthcare workers continue to work despite mental health problems [6]. Presenteeism is a significant issue, not only because of the costs associated with reduced productivity of ill workers, but also because of the potential impact on quality of care [7,8].

Mental health problems among healthcare workers are challenging because the issues are often surrounded by secrecy and silence [9]. Many workers are reluctant to admit that they are ill, and do not seek help for their mental health problems when needed. There is often a long lag time between the onset of symptoms and seeking treatment [10]. One of the risks of this delay is that issues can escalate to the point of crisis before they are addressed, causing significant social tensions in the workplace, damage to the reputation of the ill worker, and potentially compromising patient care [7,8]. Early intervention is critical to prevent the personal, social and financial costs of untreated mental health issues at work.

### Early intervention for mental ill health

There is strong evidence to support the value of early intervention and support for mental health issues. In general, providing appropriate medical treatment for mental illness at an early stage can improve outcomes including the quality and speed of recovery [11]. In the workplace, a systematic review study found that facilitation of treatment, including access to psychological interventions, significantly improved work functioning [12]. Conversely, delays in providing psychological treatment have been linked to poorer outcomes including social and cognitive decline [13]. In fact, Brouwers and colleagues [14] found that the duration of the delay in seeking help was the strongest predictor of the duration of long-term sick leave among employees with common mental health disorders. In other words, delays in seeking treatment may impede return to work if the illness leads to a mental health related sick leave.

Although early intervention is beneficial, many workers to do not seek or receive the help that they need in a timely way. According to the Canadian Community Health survey, only one third of Canadians who needed mental health services actually received them [15]. Seeking help is more likely if there is a serious mental health problem, however, several large Canadian and U.S. population-based studies reported that 15-20% of respondents would “probably not” or “definitely not” seek mental health support even if they had serious emotional problems [16]. There are many potential barriers to the process of help-seeking, but the most commonly cited barriers include perceived stigma, as well as poor mental health literacy [17,18]. Self-stigmatization and anticipated discrimination from others are associated with a reduced readiness to seek professional help for mental disorders [18]. In addition the ability to recognize mental health disorders and identify intervention options are important precursors to help seeking [17].

In addition to individual forces that affect help-seeking behavior, it is important to note that workplaces are social spaces; others in the workplace may play a key role in facilitating access to support. Managers or supervisors, co-workers, union leaders, occupational health providers and human resources personnel are a key part of the social context of work. Managers, for example, are well positioned to be a gatekeeper for needed accommodations; providing modified work, interpreting corporate policies, and facilitating access to corporate and medical resources [19,20]. Conversely, poor support from managers reportedly doubles the risk of a mental health related long-term sickness absence [21]. Co-workers are another key stakeholder group. They may be the first to notice changes in employee behaviors, and can have a significant impact on whether an employee is supported or discriminated against when they are unwell [6]. Studies in the nursing profession, for example, have revealed a phenomenon of ‘horizontal violence’ that can occur whereby nurses discriminate against colleagues who have an identified mental health problem [22,23]. Other key stakeholders in the workplace include union leaders, who can be an advocate for workers who are struggling with mental health issues, occupational health and wellness providers who are responsible for addressing employee health issues, and human resource staff who are responsible for addressing individual and organizational issues that impact work performance [24]. One of the challenges with the diverse group of stakeholders is that they have different agendas and poor communication may in fact be a barrier to employees receiving the supports that they need, when they need it [9]. Clearly there are a number of complex forces that shape whether or not employees get the help that they need in a timely way.

### Employer role in addressing mental health at work

There are increasing expectations for employers to take a more active role in addressing the mental health needs of their workers. Shain [25] makes reference to a ‘perfect storm’ in which there are mounting legal pressures for employers to provide and maintain a psychologically safe workplace, citing many legislative and legal decisions that are driving this change. In Canada, a National Standard for Psychological Health and Safety in the workplace was released in 2013, which focuses on mental illness prevention and mental health promotion in the workplace. The standard is voluntary, but is intended to provide systematic guidelines for employers that will enable them to develop and maintain psychologically safe and healthy work environments for their employees [26]. A strong business case has also been established for the value of supporting the mental health of workers. In healthcare, for example, there is evidence that the consequences of healthy workplaces are not only individual (i.e., psychological, physiological and behavioural) and organizational (e.g., decreased absenteeism, reduced turnover and improved performance), but can also have a positive impact on, quality of care and patient safety, and ultimately on society (in the form of reduced healthcare costs) [27].

The mandate to support employee mental health is clear, but there is less clarity on what programs or approaches are needed. Smith, Bielecky & Frank [28] argue that we need more information “on what factors workers want changed, what factors employers want to change, which of these factors can actually be changed and how these changes can occur” (p.67). In order to design strategies to facilitate early intervention and support for healthcare workers with mental health issues, a clear picture is needed of the forces that shape behavior. Contextually relevant research, considering the unique aspects of healthcare workplaces can help to inform program development. The purpose of this study was to explore the key forces that shape early intervention and support for healthcare workers who are struggling with mental health issues, and to identify barriers and opportunities for change.

## Methods

This study is part of a larger initiative to develop an early intervention and support program for healthcare workers who are struggling with mental health issues. A multi-phase knowledge-to-action framework was used to guide the process of stakeholder engagement and program development [29]. This paper focuses on findings from the first phase of the project in which input was gathered from employees regarding their experiences with mental ill health at work as well as their perspective regarding potential opportunities and barriers to supporting colleagues with mental health issues. A qualitative case study approach was adopted to gather input from a range of workplace stakeholders in a large healthcare organization. Case studies are particularly suited to research questions that require a detailed understanding of social or organizational processes [30,31]. Context is particularly important when trying to understand and explain behavior, and case studies provide an opportunity for in-depth examination of emergent and changing properties of organizations, including the social dynamics that unfold in workplace relationships [32]. Since the issues are not isolated from the context within which they occur, case studies can be a rich source of data that allows the researcher to retain the meaningful characteristics of real-life events [31]. Case study methodology varies, but in this study, the focus was on description and explanation of the ways in which employee mental health issues were understood and addressed in the context of healthcare work [33].

The study was conducted in a large, multi-site healthcare organization in a mid-sized urban setting in Ontario, Canada. The organization employs almost 10,000 full and part-time workers and provides services to clients of all ages, from emergency and acute care to long-term rehabilitation and palliative care. The target group consisted of a) employees within the organization who had personally experienced mental health issues, and b) employees who had not necessarily experienced mental health issues themselves, but had contact with others at work who were struggling. Ethics approval was obtained through the Hamilton Health Science/McMaster Health Sciences research ethics board, and informed written consent was obtained from all participants.

An advisory team, consisting of 12 stakeholders from across the organization was developed at the outset of the project to provide input to the researchers regarding strategies to facilitate recruitment, theoretical sampling, data collection, and dissemination of the study findings. In conducting organizational research, an advisory team is recommended to build stakeholder engagement, not only in the research process, but eventual uptake of the study findings [32]. Since the primary investigator was an outsider to the healthcare organization, the advisory team provided insights into the unique structure and culture of the workplace that might affect recruitment and data collection, and facilitated communication about the study to stakeholders across the organization. Advisory team members were strategically recruited to ensure leadership and representation from all key stakeholder groups and site locations. Each advisory team member represented employees from different social locations in the organization and had different “stakes” in the outcome of the project, but they shared a similar level of commitment to addressing mental health issues of employees. The 12 member advisory committee included two representatives from Health, Safety and Wellness, six managers and supervisors from clinical as well as non-clinical areas across all five sites, leaders from all three unions, and front-line workers, including employees who had personally experienced mental health problems. The team met eight times over the course of the year. Although they did not directly participate in the process of data collection or analysis, the advisory team meetings were an opportunity for the researchers to engage in problem solving regarding recruitment of hard-to-reach employees and how to handle the demand for participation, and to discuss potential implications and application of the study findings.

The core research team included the primary investigator, two student occupational therapists, and the director and manager of Health, Safety and Wellness. The primary investigator had a clinical background as a mental health service provider, as well as research expertise related to workplace mental health, and qualitative methods. As such, her perspective was informed by this position, and her commitment to understanding the needs of workers with mental health issues in the context of healthcare work. The two students conducted the majority of the interviews, under the supervision and guidance of the principal investigator, and also engaged in process of analysis. Research team members from Health, Safety and Wellness facilitated entry into the organization and communication with the advisory team, but were not directly involved in the process of data collection or analysis. Since the researchers were from outside of the organization, this facilitated assurances of confidentiality for study participants.

Recruitment for the initial phase of the project was facilitated through posters across the organization asking for participants who “had something to say” about employee mental health issues within the organization. In addition, information about the project was emailed to members of the advisory team, who then forwarded the information to their contacts. There was also some snowball sampling of participants recruiting others in the workplace. The purpose of recruitment was to find key informants from a range of social locations in the organization. Purposive sampling was conducted to include variation among participants in terms of job tenure, position within the organization, hospital site location, nature of work (clinical vs. non-clinical), and experience with mental health issues (personal, family or co-worker). Many of these dimensions of difference emerged naturally in the process of recruitment, and enhanced methodological rigor by providing an opportunity to examine the issues from a range of perspectives [34]. Recruitment was intended to proceed to the point of saturation, where new information was not being shared [35]. Although this point was ostensibly reached after interviewing approximately 16 participants, a second phase of data collection was added since there were many more volunteers than initially anticipated. Since engaging champions across the organization was a key goal of the project, data collection was expanded to include the voice of additional employees, by converting the interview questions to an online questionnaire.

Data collection was completed over an eight-week period in the spring of 2012, consisting of in-depth interviews as well as an online survey. In the initial phase of data collection, in-depth interviews were conducted with 16 participants, including eight employees who had personally experienced mental health problems, and eight workplace stakeholders who interacted with ill employees (e.g. union leaders, health and wellness staff, managers, co-workers). The interviews were approximately one hour in length, exploring participants’ perspectives and experiences regarding the ways in which employee mental health issues were addressed (or not addressed) in the organization, as well as their opinions about potential opportunities for early intervention. Interviews were audiotaped and transcribed verbatim.

A second phase of data collection included an online questionnaire sent to additional employees who expressed an interest in participating in the project, but could not be accommodated in the interview process. These employees were provided the link to an online survey, which was based on similar open-ended questions asked in the interview, including questions about personal experiences with employee mental health issues, barriers or challenges to addressing the issues, desired changes in the organization, and perspectives on the potential for early intervention and support. The questionnaire data complemented the interview data since it was a way of exploring the consistency of themes across a broader sample of participants. The majority of survey participants provided detailed responses to the open ended questions. An additional 67 workers participated in the online survey over a four-week time frame.

There were a total of 83 interview and survey participants. The eight interview participants who had personally experienced mental health issues were primarily female (87%), front-line employees (87%), in clinical roles (63%). The other eight stakeholder interview participants were all female, in primarily clinical areas (75%) in managerial roles (63%). Survey participants were primarily female (83%), and in front-line (42.9%) and clinical roles (34%). Just over half of all participants disclosed that they had personal experience with mental illness at some point in their life.

All of the data from the interview transcripts and surveys was reviewed in an iterative process of identifying key concepts and themes. Both of the student interviewers and the primary investigator participated in initial coding of the interview and survey data. N’Vivo**®** software was used to organize and code the data, with two independent coders for each interview transcript. Analysis was informed by a process of interpretive description [36], starting with identification of recurring concepts or ideas related to employee mental health issues, then considering patterns of behavior and the contextual forces shaping behavior. Throughout the analysis process, categories and themes were continually reviewed to ensure that they appropriately reflected the data. The final model was developed to explain the key forces shaping behaviors surrounding employee mental health issues in the organization. Credibility of the findings was supported by feedback from the advisory team.

## Results

**Figure 1:**
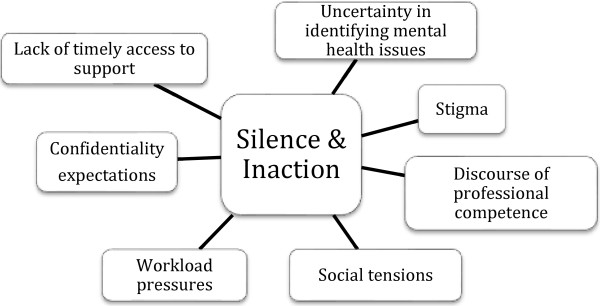
The web of silence and inaction in the workplace.

Analysis of the qualitative comments from the online surveys and participant interviews reflected a number of consistent themes regarding the ways in which employee mental health issues were addressed (or not addressed) in the workplace. Overall, many of the comments highlighted what appeared to be web of silence and inaction in the workplace surrounding employees with mental health issues. Study findings highlighted key forces that contributed to this pattern of secrecy and inaction; from uncertainty in identifying mental health issues through to the lack of accessibility to timely supports (see Figure 1). In presenting the study findings, a description of the overall pattern of silence and inaction will be followed by an explanation of each of the forces shaping this pattern. Finally, exceptions to the recurring pattern will be considered, using quotes from the study participants to illustrate key points

### The web of silence and inaction surrounding employee mental health issues

Participants shared a range of experiences related to mental health issues at work, including personal struggles as well as challenges with colleagues, supervisors and family members. Many emotional stories were shared regarding addictions issues, anxiety, mood disorders, bullying, and even suicide. Although diverse stories were noted, a common theme in many of the stories relates to frustration and concerns that mental health issues were not adequately addressed. For workers who personally experienced mental health issues as well as their co-workers and managers, the study appeared to serve as a forum for venting their frustration about inadequacies in the system and lack of support for workers who were struggling with mental health issues.

The majority of participants reported that mental health issues were surrounded by secrecy and silence, and that employees did not reach out or act when an employee was struggling. There were many reports of issues that were hidden, ignored, and/or mislabeled, by workers themselves as well as by the people around them. Some workers, for example, talked about maintaining a façade of normalcy even though they were struggling inside. Others talked about the lack of response from others when issues were disclosed. One worker explained; *“They don’t want to hear about it. They just want to know that everything is fine. As long as you don’t talk about it, you know, everything is working out fine. And that is not true”.* Another employee lamented; *“I’ve been called into the office and told that I can’t be upset at work”.* Rather than discussing issues directly, there were many reports of rumours and gossip about workers who were struggling with mental health issues. In the words of one stakeholder; “*It felt to me like a bit of a ‘non-talked about’, but ‘talked about’ issue that was very awkward*”. Innuendo and rumours replaced direct, open discussion.

It was noted that the secrecy and silence surrounding mental health issues was ironic in many ways. One of the participants remarked that “*we are in a caring profession and we don’t care about our own co-workers”.* There seemed to be a number of tensions experienced by employees in relation to recognizing mental health issues and then reaching out to offer or seek help for either oneself or a colleague who may be struggling with mental health issues. The tensions in responding appeared to be multi-layered, reflecting not only a lack of knowledge about mental health issues, but attitudinal and organizational barriers to reaching out to provide or seek help.

### Uncertainty in identifying mental health issues

The first area of tension relates to awareness of employee mental health issues, with many participants expressing uncertainty about whether there is an illness that should be noted. Several participants talked about subtle changes that may be difficult to detect, particularly at first. Unlike a physical injury, mental health issues were not as easily recognized. As one worker explained; *“I think there’s still that… idea where you know mental health [problems] means something like schizophrenia and things like that. It’s not just the subtle things that you don’t normally see. So I think that a lot of time it gets looked over because it’s not as overt.”* Another participant remarked that everyone reacts differently, with some who are tearful, others who appear angry and others who withdraw from others. One participant reflected on the challenges of knowing when to respond, “*What things should you be worried about? … What’s part of personality where people have ups and downs?*” Since a spectrum of issues is possible, they may not be immediately identifiable as a mental health concern. If difficult behaviors are normalized as part of the employee’s character, the tipping point as to when action is needed is unclear. One of the program supervisors reflected on subtle changes in how an employee talks or acts, emphasizing the need to pick up on these cues as part of a pattern of behavior change over time. From her perspective, co-workers or front-line managers are in the best position to identify these subtle changes, but may not have adequate knowledge to do so. Another identified challenge is the time and energy it takes to notice changes in colleagues. One participant remarked that staff *“become so involved in their work and don’t notice their colleagues struggling”.*

Identification of employees who are struggling seemed to be complicated by the fact that many workers experience stress and find it difficult to cope. As a result, there was some normalization of the experience. “*There’s a spectrum - we can all feel down and not want to get out of bed, but we force ourselves to do it. Where on the spectrum do people fall when they start to become diagnosed and how do we differentiate?*” In some cases, participants talked about reluctance to label or diagnose mental health problems in others. One participant, for example, argued; “*I don’t think it is appropriate for staff to play doctor and try to diagnose their coworkers with mental illnesses*”. A supervisor expressed similar discomfort, “*it just feels this whole sense of ‘unsure’ and maybe it’s not necessary to try and label it or do anything other than describe the behaviors and let them work out what’s going on…”* Overall, the uncertainty about how to identify mental health issues and interpret behavior change in the workplace was a recurring theme.

### Stigma and shame surrounding mental health issues

A second source of tension relates to the stigma of mental illness that shaped not only the actions of workers struggling with their own mental health issues, but the actions of their colleagues as well. The stigma associated with mental illness was reported by many participants to be a significant force that affected whether employees were motivated to seek help for themselves. Workers who struggled with mental health problems explained that they were reluctant to disclose to others or seek help because of the fear of stigma and discrimination. In some cases, it was internalized stigma that prompted the silence; *“Sometimes you feel ashamed… so you don’t want to disclose any of that negative information”.* In other cases, there were concerns about how it might affect one’s reputation with others; *“My credibility as a therapist… would be compromised if people knew about it”.* Workers shared a number of examples where their fears of stigma and discrimination did in fact occur in the workplace. As one participant reported, “*I had one of my co-workers say to me ‘well, you seemed fine that afternoon’. …Because they didn’t see it, they didn’t get it. And I felt judged”.* Another participant expressed similar concerns; “*many people do not recognize these types of illness as a true illness*”, and as a result “*absences due to these illnesses are almost always met with disdain, rumour and attitude*”. They felt as if the legitimacy of their mental health problems were questioned.

The stigma associated with mental illness was reported to be a significant barrier to reaching out to offer support to a colleague. Many examples were shared regarding employees who were negatively labeled once their issues became public. Interpretation of changes in an employee’s mental health varied, from simply minimizing the problems to dismissing them as “bad behavior” or poor performance. In some cases, workers were labeled as malingering or simply not doing their work. Dismissing ill employees as “*lazy*” or “*crazy*” served to absolve colleagues from responsibility for providing assistance. Several employees talked about the need for education to reframe attributions from one of blame to one of support. One of the participants argued passionately about the need to change attitudes in the workplace:

…to come up with other explanations for someone’s behavior rather than ‘oh, they’re just lazy or incompetent at their job’ , or making a personal judgment of them. Like okay, maybe something else is going on here, maybe we should give them a bit of a break or something like that.

These comments reflect the pervasive role that stigma plays in supporting employees who are struggling with mental health issues.

### Discourse of professional competence

One barrier that seemed to be specific to the healthcare environment, relates to what seemed to be an image or expectation of healthcare providers to be invincible and therefore able to cope with the stressors that are put upon them. One employee, for example, stated; “*I think that it is frowned upon for health care workers to have any kind of mental health issues since we are responsible for people's care”.*

This discourse of competence reportedly affected not only whether employees sought help for themselves, but also whether they offered help to their colleagues. For example, some employees tried to be stoic and did not seek assistance when they felt overwhelmed. One participant explained; “*As healthcare providers, we spend so much time just ‘sucking it up’ emotionally to do our job, that we often don’t take care of ourselves properly”.* This expectation to be invincible also led to an attitude of intolerance for workers who are struggling. Another worker, for example, complained “*there is little to no empathy, but rather a sense that if you can’t cope, get out of the profession”.* A manager reported that he used to say to his employees; “*You’re stressed? … Get over it”.* The prevailing discourse seems to be related to a responsibility to maintain an image of professional competence, rather than disclosing any need for support.

### Social tensions

Social tensions associated with breaking the silence and acting upon concerns for colleagues was another key theme reported by many participants. Reluctance to reach out for help or offer help seemed to be compounded by social barriers, such as fears about how the ill employee might respond to an offer of support. As one worker mused, “*I can see how it might backfire and that person might be ‘no, I’m not that way’…*” Workers who denied that they were ill made it difficult for others to reach out to offer support. There were fears that the worker could be upset or potentially volatile in response to concerns expressed by others. Many participants shared examples of relationships that were strained in the workplace by employees who denied that they had mental health problems. In some cases, there was a power differential, where a supervisor or physician was displaying signs of a mental health or addiction issue, and the fear of retribution compounded the reluctance to act. In other cases, inaction was attributed to a reluctance to “create trouble” for the person who was ill. The following example illustrates the reluctance of a union representative to act upon concerns about an employee drinking problem;

“Over time she was coming in late for lunch, after lunches and someone was saying they were smelling alcohol on her; there were lots of whispers. I went to the union rep who was actually working with us and she would say ‘sh, sh, sh’. She didn’t want to hear about it because she’d probably have to report”.

There were several cases where participants described situations where an employee was struggling to the point of not being able to perform his/her job competently. The employee did not recognize the problems, and as a result, the co-workers felt obligated to report the worker to his/her professional college for disciplinary action. They were very aware of their duty to report, but resisted acting because of the significant social repercussions that this would have on their relationship with their colleague in the workplace. The process was described as extremely stressful for all involved. Silence and inaction therefore was a strategy to avoid dealing with the social tensions associated with acting on their concerns.

### Workload pressures

Another key contributor to the reluctance to reach out relates to workload demands. Many participants talked about feeling overwhelmed with their own work, leaving little energy left to look after their colleagues. As one worker explained; “*It’s very challenging, especially when you are already dealing with a very, very heavy workload. … Sometimes it’s just too much. People have too much on their plate. They just can’t do it”.* Taking responsibility for a co-worker was seen as an added stress in an already stressful, demanding work environment. *“I suppose if we see coworkers struggling, we could try to talk to them earlier, but it’s hard on a busy day”.* One of the participants cautioned against taking on this extra responsibility, feeling that it could pose a mental health risk to colleagues expected to provide the support: *“It is arguably exhausting to spend your day caring in a professional role and then spending your off time (lunch time, breaks) caring in an informal role. I think that’s not a good scenario for other staff”.* Several participants talked about feeling conflicted between their desire to help, and their responsibility to meet work demands.

“…you feel compelled and want to be supportive of that person, but as a clinician you have… a need to get work done - and needing to rely on someone and not always feeling like I could do that”.

On the one hand, some workers had a sense of compassion and a desire to help, but on the other hand, there was concern that an ill colleague may interfere with their own mental health and ability to perform at work and provide quality care. This tension made it difficult to know when and how to respond to colleagues who were struggling.

### Confidentiality expectations

Another organizational issue reported by many participants relates to expectations regarding disclosure. Many participants commented on issues of confidentiality and disclosure related to employee mental health issues. Since it was a healthcare setting, maintaining confidentiality regarding client medical information was a key organizational policy. Although not as explicit as the client confidentiality policies, a number of participants made reference to the importance of maintaining confidentiality regarding employee mental health problems. The importance of confidentiality was stressed in light of the stigma associated with mental illness. Several participants explained that they were reluctant to share information about themselves or others because of fears of stigma and discrimination.

Although confidentiality was recognized as important, there were participants who expressed concerns about the tensions that the secrecy created. One participant, for example, suggested that there should be some way of talking about issues in an effort to support employees who are struggling:

“*Some kind of disclosure that doesn’t threaten the person’s autonomy or confidentiality, but allows co-workers to understand the person’s situation” “How can you ask an employee if they need help? Better yet, how can you tell an employee that they need help??? I thought there were confidentiality issues about that”.*

Several participants, particularly those in management positions, felt that the mandate for confidentiality was restrictive at times since it held them back from reaching out to employees who were struggling. As one manager reported; *“I know that there’s reasons not to know stuff about a person’s medical status, but when you’re trying to work with them in other ways, it can really tie your hands”.* Managers felt that they received very little information, and that this made it difficult to respond to the unique needs of the employee. In addition, front-line employees felt that they needed more information from managers. One participant, for example, complained; “*I know they [managers] are bound by confidentiality, but it would be nice if they could tell the other staff something basic…”* There were several comments about the challenges associated with rumours and innuendos that emerge when issues aren’t shared directly; highlighting that the lack of open communication actually perpetuated stigma within the organization.

### Timely access to mental health supports

Another key tension related to mental health supports at work concerns the difficulties that many employees experienced in being able to access the supports they needed in a timely manner. Several employees who struggled with mental health issues explained that it would be helpful to have onsite support when they were feeling stressed. Even just a 20-minute conversation with someone they felt would be helpful in “offloading” their stress, and prevent problems from escalating. Others expressed concern that they were not able to attend therapy appointments during work hours. One employee, for example, said that her request for time to see a psychologist was refused, with little explanation, other than “*I’m told we don’t have staffing and work always comes first*.” In some programs, there was no coverage for employees who were off, making it difficult to take the time for medical appointments during work hours. Instead, some employees reported taking vacation time to attend therapy appointments. Although the employee assistance program (short-term counseling services provided by the employer) was described as a very valuable service that offered appointments outside of work hours, other options for mental health support were limited.

### Exceptions to silence and inaction

Although there were clear and repeated examples of barriers to support for workers with mental health issues, it is important to note that there were a few exceptions to the typical pattern of silence and inaction. These exceptions or “negative cases” were examined in terms of how they could inform emerging findings about help seeking and outreach behavior [37]. Examples of exceptions included specific individuals, the culture of particular teams, and organizational policies.

Supportive individuals.

Several participants talked about individuals, typically managers or supervisors, who were intuitive and supportive of employees who were struggling. One employee praised a particular supervisor who communicated genuine caring and concern. If the employee was struggling, she felt that she could approach him/her, whereas this was not the case with her other supervisors. One of the managers talked about his personal struggles with mental health problems and how this actually made him more sensitive to the issues of others.

*“My experience [with mental illness] has made me a better manager, that’s the shocking part of all of this for me. I’m not as cocky as I used to be. I think I’m a little more calming in dealing with performance issues from a staff perspective than I would have been before*”. *I’m more aware of staff who are changing from day-to-day and I will actually draw them into a discussion to say ‘hey, what’s going on?*’”

Another participant explained how she often advocated with her colleagues for a more compassionate perspective towards employees who were struggling. For example, she stated that “*there are times when somebody might come in and [say] ‘oh, so and so lost her mind’*.” Her response would be to explain that the employee is simply going through a really hard time. She felt that her efforts to “*diffuse”* the situation helped her colleagues to realize, “*okay, I’m not the target, it’s just what they are going through right now*”. These examples illustrate ways in which employees tried to challenge the culture of blaming employees who were struggling.

Supportive teams.

In addition to examples of individuals who were supportive, a couple of participants talked about the unique culture in their team. For the most part, participants felt that there were negative responses to employees with mental health problems, but there were a few exceptions, where participants described a culture of teamwork and support. Many participants talked about the importance of creating a culture of support in the organization to create a proactive response to mental health issues.

“I think if people said ‘you know what, I’m feeling down, but I know in my corporation it’s freely acceptable and the supports are in place’, you feel comfortable bringing forward the information that you’re not doing well because you know it’s going to be supported”.

The importance of changing the status quo was echoed in the comments of several participants. There seemed to be recognition that although there may be good intentions, this does not always translate to positive action. One participant talked about the importance of gaining *that compelling need to help a co-workers as much as you would a patient,* and another lamented that there may be “good intention” to provide help, but it often is not done in a timely or skilled way. The opportunity to disclose without fear of judgment or retribution was viewed as a critical step in the process of proactively seeking and receiving support.

Organizational policies.

There were several policies identified by participants as triggers for dialogue and action related to employee mental health issues. One of policies identified by several participants was the attendance management policy. Although it triggered dialogue and outreach, a number of participants specifically mentioned that the current policy was problematic. The policy, not unlike those in other organizations, outlines procedures for managing employee attendance, including guidelines that should be followed if an employee is absent for a certain number of days. According to the policy, supervisors are instructed to meet individually with employees who are absent for a specified period. Several employee participants complained that the policy was punitive and unsupportive for workers who were struggling. One participant, for example, explained that the process actually exacerbated her difficulties:

“I get an email from her [supervisor] saying okay, your absence rate is up this percentage, we need to have a meeting. Now my anxiety is through the absolute roof cause now I’m thinking, oh my God, I’m going to lose my job and then it makes it worse and then I come into work and I can’t cope again. It’s like –that’s not very helpful”.

Similar concerns were voiced by other employees regarding the apparently punitive nature of the attendance management program. As one participant recommended; “*You have to look at it in more of a broad perspective. People aren’t numbers. Yes, they can take time off and they may take an extended time off, but you have to look at the reasoning behind it”.* Instead of creating a policy where the employee feels guilty for taking time off, the participant emphasized the importance of constructive problem solving and support. The policy reportedly had a negative way of shaping when and how sick leave was addressed. The other policy, mentioned earlier, relates to reporting mental health issues to the professional college of the affected employee. As stated earlier, there were several instances where this action was taken and it was perceived to be stressful for all involved. One of the participants was on the receiving end of this reporting process and commented on how demoralizing it was for her. She felt that the professional colleges discriminate against healthcare workers with mental health issues.

Overall, the study findings revealed many barriers to dialogue and support for employees with mental health issues. Although there were several exceptions to the rule of silence and inaction, these actions were often isolated and in some cases were perceived as punitive rather than supportive.

## Discussion

It is evident that there were many complex barriers that interfered with participants getting the help that they needed for their mental health problems. The pathway to early intervention and support seemed to have many hurdles to overcome, from lack of recognition of the signs of mental ill health, to stigma, interpersonal tensions, and a discourse of professionalism that prevented employees from taking responsibility to provide or seek help. These individual and social barriers in turn were accentuated by work demands and time pressures, in addition to confidentiality expectations, unsupportive policies, and lack of timely access to mental health supports. An understanding of these multi-layered forces is critical to developing strategies for organizational change.

The first theme regarding uncertainty in identifying mental health issues appears to reflect the need for mental health literacy training. Mental health literacy has been defined as a combination of knowledge and beliefs that contribute to the recognition, management or prevention of mental disorders [38]. Jorm [17] explains that mental health literacy consists of several components, including the ability to recognize specific disorders or different types of psychological distress, as well as knowledge and beliefs about intervention options. There are a number of mental health literacy programs that have been developed and implemented in workplace settings in an effort to overcome knowledge barriers to action. Programs include education of high-level managers as well as front-line workers, and are typically offered over one or two sessions [39,40]. Mental Health First Aid, for example, is a standardized mental health literacy program that has been widely implemented and evaluated, including several workplace studies [41]. There is emerging evidence supporting the impact of literacy training approaches on awareness of how to identify and respond to mental health issues at work [39,41]. Despite the reported success of literacy programs, it is important to note that increased knowledge does not necessarily produce supportive attitudes or behaviors. In fact, healthcare providers have been criticized for their stigmatizing attitudes towards people with mental illness, even though their mental health literacy levels are ostensibly higher than in the general population [42]. The uncertainty expressed by participants in this study does not necessarily reflect a lack of knowledge of the symptoms of mental illness; instead, it may reflect differences in how these signs are interpreted in the context of day-to-day work. In some cases, mental status changes in colleagues were noted, but the tipping point for action was not clear. Attributions for the changes ranged from normalizing or minimizing the issues to criticizing the ill employee for his/her behavior. The way in which the situation was interpreted seemed to be shaped by the personal pressures experienced by workers, negative or judgmental attitudes, and beliefs about whether it was appropriate to intervene. The personal and social context must therefore be considered in educating healthcare workers about how to recognize and respond to workers who may be struggling. Mental health literacy in the context of healthcare work seems to require a specific lens that goes beyond simply identifying the signs and symptoms of mental illness.

The second theme regarding the reluctance to take responsibility for seeking or offering help underlines the need to address the attitudinal and interpersonal barriers to action. Negative attitudes towards mental illness and employees who were struggling seemed to be a significant barrier to reaching out to offer help. This finding is consistent with the research documenting how the stigma associated with mental illness is a significant barrier to seeking help and to providing help to colleagues who are struggling [18,43]. In order to address stigma and negative attitudes associated with mental illness, contact-based education is recommended as a best-practice approach [44]. Contact-based education is a knowledge translation strategy that creates opportunities for positive interpersonal contact with someone who has personally experienced mental health issues [45]. Key ingredients for contact based education include voluntary, positive, prolonged contact with a respected peer of equal status [46,47]. Evaluation studies of contact-based education with a range of student groups have reported positive outcomes, including a significant reduction in prejudice and social intolerance [45,48]. Additional research is needed to examine how contact-based education might be implemented in a healthcare workplace however it does show promise as a strategy to address attitudinal barriers.

Reluctance to take responsibility that was evident in this study was not only linked to stigmatized beliefs, but also to social and organizational disincentives to action, including a professional discourse of invincibility, fears of retribution, and day-to-day work demands that leave little time or energy for compassionate support. The professional discourse of invincibility is congruent with traditional views about the service provider who is supposed to be an objective, detached expert with social distance from the problem of illness [49]. Overcoming deep-seated values about the invincibility of healthcare providers may be challenging. There is evidence, however, that resistance to the prevailing discourse has the potential to evolve over time with public recognition of positive role models in healthcare who admit to personal vulnerability, as well as acknowledgement at an organizational level of the importance of support for the mental health of healthcare employees. There is some emerging literature, for example, documenting healthcare providers who have disclosed their personal experiences for the purposes of addressing the stigma associated with mental illness [50,51]. Another study conducted in the UK documented the potential of workplace-based champions to facilitate culture change within an organization related to promoting mental health and well-being [52]. The UK study emphasized the importance of internal champions who can raise awareness, build partnerships and encourage changes to work procedures. Supervisor training programs also emphasize the importance of leadership in promoting change in how issues are addressed [40]. Finally, leadership is needed at the highest levels of an organization, to communicate the importance of the issues, allocate sufficient resources for meaningful interventions, and demonstrate organizational commitment to change [53].

In order to address other interpersonal and work-related barriers to action, incentives might be needed for reaching out to provide or seek help. These incentives (e.g. positive recognition and support from supervisors) need to outweigh the social risks and personal costs of a proactive, compassionate approach [54]. There is an interesting, emerging body of literature on “organizational citizenship” that casts light on forces that may influence whether or not an employee reaches out to a colleague who is struggling. Organizational citizenship, a term that originates in the management literature, refers to behavior that is “above and beyond” the job description of the employee, and contributes to the effective functioning of the organization [55]. Helping behavior is one of the key dimensions of organizational citizenship. Research on the antecedents of helping behavior includes high team morale and supportive leadership as conducive to altruism or outreach to others [54]. There can be a cost to reaching out in terms of time and emotional energy therefore social and organizational supports need to be in place to facilitate this process.

The third theme relates to organizational structures and policies that shape action. This theme reflects the importance of not simply focusing on changing employees, but considering the organizational structures and policies that affect behavior. Although policies are often designed to be supportive, they are not always perceived as such and may in fact create barriers or additional complications for employees and their colleagues. Golden [56] notes that one of the challenges in healthcare is that there are competing interests at times, particularly due to the combination of business and human service delivery perspectives. Hospitals can be highly bureaucratic, with an emphasis on institutional efficiency that may conflict with the supportive focus of a people-processing organization [57]. The study findings highlighted some of the interpersonal tensions that can be created in part, by competing priorities related to the mandate for productivity and performance versus the mandate to support workers who are struggling. For example, the attendance management policy is a way of identifying employees who are frequently ill and potentially in need of additional support, however, the policy could also be perceived as a way for employers to pressure employees to stay at work, thereby reducing the costs associated with sick leave. Some policies seemed to reflect differing messages about when and how to break the silence and take action when an employee is ill. For example, confidentiality policies restrict dialogue and provision of supports, as do inadequate accommodation procedures. Attendance management and professional competency reporting guidelines trigger action, but often at a point where there is an emerging crisis that may be difficult to avert. Explicit guidelines and resources to promote early intervention and support are therefore needed to prevent a mental health crisis, as well as prevent irrevocable damage to workplace relationships. In addition, mental health supports need to be readily available to employees who need them. Current initiatives to promote psychological health and safety in the workplace outline strategies for employers to create and implement workplace policies for the purposes of mental health promotion and illness prevention [53].

### Study strengths and limitations

One of the strengths of this study is that it captures the complexity of issues related to early intervention and support for healthcare workers who are struggling with mental ill-health, including consideration of a range of complex forces that shape behavior. Inclusion of input from over 83 workers across the organization as well as triangulation of data sources (multiple stakeholders) added both depth and breadth to the study findings. The findings therefore reflect a nuanced understanding of how silences are produced and reproduced in the context of healthcare work. This understanding is an essential building block to developing effective and relevant approaches to intervention.

One of the study limitations is that it was conducted with a cross-section of employees in one healthcare organization. Study participants were volunteers and do not necessarily represent the viewpoints of all individuals across the organization. In particular, many of the participants were female, spoke English, and worked in clinical positions. Although the majority of employees in the organization meet this description, there may be important viewpoints that are missing (e.g., male employees, workers who do not speak English, and those who work in non-clinical areas). It should also be noted that the issues could differ in workplaces that are smaller and/or have a different set of personnel, policies and practices with respect to workplace mental health. Nevertheless, the findings point to some key issues that need to be considered across healthcare workplaces. It should also be noted that this study was conducted in a Canadian healthcare context. As such, some of the forces related to workplace structures and organizational policies may be unique to the context of the Canadian healthcare system. Although there is evidence that stigma, silence and reluctance to seek help are an issue among healthcare providers in other industrialized countries [2,6], additional research is needed to explore whether the unique forces identified in this study are applicable in other healthcare settings.

## Conclusion

Overall, the study findings highlight the need for a multi-level, multi-pronged approach to early intervention for healthcare workers. To date, many workplace programs adopt a uni-dimensional approach, and focus almost exclusively on building literacy [39,58,59]. As a result, important attitudinal and organizational barriers may be missed. Top-down approaches that consider organizational structures and policies are important to build a culture of support for psychological health and safety [53]. Bottom-up approaches are also needed, however, to build a culture of compassion and peer support. Ultimately, a multi-layered strategy that incorporates contextually relevant ways of addressing the unique features of healthcare work will have the greatest impact on supporting the mental health of workers and prevent the consequences of mental ill health. Further research is needed to track the relative impact of various approaches in order to effectively unravel the web of silence that surrounds the mental health of healthcare workers.

## Competing interests

The author declares that she has no competing interests.

## Authors’ contributions

SM was the principal investigator for the research; she designed the study and coordinated all stages of data collection and analysis. SM was also the sole author of the manuscript.

## Pre-publication history

The pre-publication history for this paper can be accessed here:

http://www.biomedcentral.com/1471-2458/14/138/prepub
